# The Relationship between Just World Beliefs and Life Satisfaction

**DOI:** 10.3390/ijerph17176410

**Published:** 2020-09-03

**Authors:** Warren G. Harding, Jasmin Tahmaseb McConatha, V. K. Kumar

**Affiliations:** Department of Psychology, West Chester University of Pennsylvania, West Chester, PA 19383, USA; warhar93@gmail.com (W.G.H.); kkumar@wcupa.edu (V.K.K.)

**Keywords:** belief in a just world, life satisfaction, self, others, distributive, procedural

## Abstract

An important and often unexplored factor shaping life satisfaction is one’s perception of the world as a “just” place. The “just world hypothesis” is predicated on the idea that the world works as a place where people get what they merit, an idea that often serves as a means for people to rationalize injustices. The research addressing just world beliefs has expanded into a four-factor model that categorizes just world beliefs for self and others into subcategories of distributive and procedural justice. Distributive justice involves evaluations of the fairness of outcomes, allocations, or distribution of resources, while procedural concerns evaluations of the fairness of decision processes, rules, or interpersonal treatment. This study explored the relationship between the four just world beliefs subscales and overall satisfaction with life and examined their associations with demographic variables including ethnicity, age, gender, religion, and social class. The relationships of demographic factors with justice beliefs and life satisfaction generally yielded very small effect sizes. However, respondents who identified themselves as middle and upper class reported higher levels of life satisfaction than those who identified themselves as lower class, with a medium effect size. Consistent with the results of earlier research, regressing life satisfaction on the four justice beliefs subscales indicated that the two self-subscales (distributive and procedural) were significantly predictive of life satisfaction, but the two other subscales (distributive and procedural) were not.

## 1. Introduction

The belief that in life people get what they deserve is widespread. Believing that the world is a just place enables people to tolerate and cope with injustices experienced by themselves and others. Such a belief can rationalize an acceptance for social inequality, lack of access to health care, poverty, and the mistreatment of others by the system. Holding a view of the world as just or unjust can also lead to social activism and motivate people to take action, as the recent “Black Lives Matter” movement has done in the United States. It is possible that believing that the world is fair and just will likely lead to less anxiety and greater life satisfaction.

### 1.1. Just World Beliefs

Belief in a just world (BJW) is predicated on the idea that the world is a just place and that people get what they deserve. This belief can also serve to rationalize perceived injustices [1,2]. People rely on the comfort of the belief in a “just world” and understand their own and others’ experiences accordingly. There are, however, personal and social implications associated with such a view. Correia and Dalbert [3] discussed three primary functions of just-world beliefs: they (a) influence members of a society to act fairly believing that they, in turn, will be also be treated fairly; (b) promote greater social trust and cohesion; and, (c) provide a way to assign meaning to one’s life circumstances. The world can be understood as “just” for oneself (BJW-self) or others (BJW-others). BJW-self is associated with greater well-being, and BJW-others is associated with social and cultural connections [4]. BJW-others has been found to have a negative association with altruistic behavior, and BJW-self is correlated positively with altruistic behavior [5]. Bartholomaeus and Strelan [6] found BJW-self to be positively correlated to forgiveness.

In the last decade, two other justice beliefs have been added to beliefs about self and others: distributive and procedural justice [1]. Distributive justice beliefs concern evaluating the fairness of outcomes, allocations, and distribution of resources, and procedural justice concerns evaluating the fairness of decision processes, rules, and interpersonal treatment [1]. Lucas et al. [7] indicated that support for policies that restrict immigrants is exclusively associated with belief in distributive justice for others (i.e., thoughts about fair outcomes for other people). More specifically, the tendency to believe in distributive justice for others was found to be associated with greater support for a policy proposing to further restrict immigrant job seekers’ capacity in the United States. Moreover, priming thoughts about justice in a sample of U.S. police officers increased their support for a policy that mandated stricter policing of illegal immigration. A strong belief in distributive justice for others appears to lead to potentially greater support for discriminatory policies.

Belief in a just world can also lead to a culture of victim-blaming. Stronger beliefs in a just world have been found to be associated with viewing social inequality, prejudice, and poverty as deserving or as a result of a lack of hard work or effort [8]. Rationalizing that people, in general, deserve what they get reinforces the belief that the “world”, in general, treats people justly [9]. Such rationalization has widespread consequences; for example, it can affect how people view those with disabilities or disorders. Rüsch et al. [10] found that such beliefs may enable people to hold prejudices and stigmatize those with disabilities. Such views can also foster prejudice against those who are victimized and oppressed—that they somehow “got what they deserved”. Studies have identified five independent sources to which people ascribe arbitration of justice in a just world: God, nature, other people, self, and chance. These sources are generally construed as responsible for allocating justice and for shaping perceptions and responses in the face of unjust events [11]. A person might rationalize that the “unjust” treatment being received is somehow deserved.

Religion, or spirituality, is also associated with viewing the world as a just place. Jost et al. [12] found that “religion provides an ideological justification for the existing social order, so that prevailing institutions and arrangements are perceived as legitimate and impartial, and therefore worthy of obeying and preserving” (p. 1). They found that religion often serves the epistemic (achieving predictability and control), existential (managing anxiety, fear, and threat), and relational (affiliating with others and group solidarity) needs of a population. Those with high religiosity might, therefore, be less open-minded, less tolerant of ambiguity, and have a higher need for control. Religiosity may, therefore, be associated with higher belief in a just world by providing a comforting, stress-reducing function, and making people feel more satisfied and/or tolerant of injustices. Reviewing the work of Marx [13], Jost et al. noted that church attendance and religious convictions blunted “support for civil rights” despite the efforts of Martin Luther King (p. 21); their results suggested religiosity is positively correlated with system justifying, rather than system challenging, views.

Personal demographic factors such as ethnicity, socioeconomic status, gender, and age may also be related to the view that the world is a just place. Hunt [14], comparing African Americans, Latinos, and Whites, found that Latinos indicated the strongest beliefs in a just world, and African Americans the weakest. More importantly, significant differences were also found by sex and socio-economic status (SES), with the greatest support for just world beliefs found among men and those with low SES. Hunt, however, noted that the effect of being Latino was reduced to about half (although still significant) when SES was included in the regression model, suggesting that the variance associated with being Latino was largely due to SES, i.e., “the inverse effect of SES among Latinos appears to be disproportionately a function of relative support for the idea of a just world among lower-status Latinos” (p. 338). In contrast, “the inverse effect of SES among Blacks is due more to the higher-status class blacks rejecting the belief in a just world than to lower class viewing the system as legitimate” (p. 338). The finding concerning the inverse relationship between SES and justice beliefs led Hunt to suggest that just world beliefs may help some people adapt to difficult social and economic living circumstances.

### 1.2. Life Satisfaction/Subjective Well-Being

Life satisfaction is an essential component of overall well-being. It focuses on the way people evaluate their lives and how they feel about their opportunities for the future [15]. Gaining an understanding of life satisfaction involves continuous assessment of how individuals view their lives as a whole, rather than focusing on current attitudes and feelings. Life satisfaction is determined by the intersection of multiple influences including income, education, health, relationships, stress, neighborhood, community, and culture [16].

Well-being (SWB) is a multidimensional construct, influenced by personal, social, and cultural factors [16]. Health and social relationships have beneficial effects on SWB, and interventions can facilitate SWB. Studies have also identified a positive relationship between religiosity and life satisfaction [17]. Some research suggests that religious people are more satisfied with their lives because of the relationships between religious attendance and community. Kahneman and Deaton [18], in an analysis of more than 450,000 responses to the Gallup-Healthways Well-Being Index and its relationship with income, found that income and education were connected to emotional well-being (emotional quality of an individual’s everyday experience) and life evaluation (thoughts people have about their life when they think about it). Income and education were more closely related to life evaluation, with emotional well-being rising with income to a certain point, beyond an annual income of USD ≈75,000 [19,20].

A global study by Morrison, Tay, and Diener [21] found that national satisfaction was a positive predictor of life satisfaction, with the relationship moderated by household income, household conveniences (e.g., appliances), residential mobility, country GDP per capita, and region (Western vs. non-Western country). National satisfaction was also related to greater life satisfaction. The results indicate that in more individualistic, well-off societies, people tend to rely more on factors such as personal health, social support, and living conditions in judging life satisfaction. In more collectivist cultures, on the other hand, people rely more on social conditions as a measure of life satisfaction. Gerstorf et al. [22] examined the role of age, social orientation, and social engagement with life satisfaction. They relied upon social variables at the behavioral level (self-ratings of social participation) and the motivational level (valuing social and family goals). The researchers applied single and multiphase growth models on 27-year annual longitudinal data from 2910 deceased participants of the nationwide German Socio-Economic Panel Study. Their results suggest that leading a socially active life and prioritizing social goals in late life were associated with higher late-life well-being, less pronounced late-life decline in life satisfaction, and later onset of terminal decline in physical health.

### 1.3. The Relationship between Just World Beliefs and Life Satisfaction

Lucas et al. [23] discussed just world beliefs and its’ relationships with various aspects of life including, but not limited to, income, health, and family, and life satisfaction, stressing that it is important to examine satisfaction in various life domains, an approach known as the bottom-up theory [24]. The bottom-up theory in relation to just-world beliefs suggests that people are more likely to support the notion of a just world when they are satisfied in various life domains. Lucas et al. [23] suggested that the tendency to believe in the prevalence of justice is associated with personal happiness and well-being. They found individual-level beliefs in justice for both self and for others to be more strongly associated with life satisfaction and health when people expressed strong beliefs in justice for others. Higher levels of individual-level beliefs in distributive justice were more strongly associated with self-rated health in high distributive justice climates and in low procedural justice climates. These cross-level interactions suggest that higher-order justice climates may moderate the relationships between individual-level justice beliefs and personal well-being.

Lucas et al. [2] found the two self-justice subscales to be more strongly correlated with life satisfaction than the two other subscales. They also found the two self-justice subscales, but not the two other subscales, were predictive of life satisfaction in a multiple regression analysis. These results were replicated in cross-cultural studies. Across all four cultures, the correlations of BJW subscales with life satisfaction were somewhat higher for the two self-subscales than the two other subscales. When life satisfaction was regressed on the four BJW subscales, the analyses revealed distributive justice for self (DJ-self) was significantly predictive of life satisfaction in all four cultures, but procedural justice for self (PJ-self) was predictive only in Canada and China. The two other scales were not predictive of life satisfaction in any of the four cultures. The results suggest that justice for self with its emphasis on personal identity and pro-self-values may relate to well-being. Whether or not age shapes views of the world as a just place is an important area of investigation. Age certainly has been shown to influence personal and social views shaping life satisfaction [25].

The studies cited above support a relationship between views of the world as a just place with well-being and life satisfaction. This study explored this connection with a further analysis of views of the world as a just place for oneself and for others both distributively and procedurally. The study also explored the relationships between just world beliefs, life satisfaction, and demographic factors including White/non-White racial identity, age, self-reported social class, gender, and religious affiliation. Based on a review of literature, it would appear that individuals from a self-perceived higher social class were more likely to view the world as a just place for the self and for others on both distributive and procedural beliefs. The study also explored attitudes regarding religion and spirituality, as well as just world views and life satisfaction.

## 2. Method

### 2.1. Participants

Volunteer participants were recruited via the snowball sampling method from undergraduate psychology classes, a local church, a senior center, and a social networking site. Participants ranged in age from 18 to 80 (with a mean age of 40). Surveys were administered individually, in small groups, or posted on a personal social media site to be completed anonymously by volunteers. A total of 186 paper surveys and 115 online surveys were completed for a total number of 301 participants. The overall sample (*n* = 301) consisted of 171 females and 130 males.

Education levels ranged from high school to advanced degrees, with most participants having some level of higher education. The participants were divided only into two categories, White (*n* = 202) and non-White (*n* = 99), because few respondents identified themselves in other ethnic categories. This division is reflective of U.S. national demographics [26]. Participants were divided into social class categories based on their self-reported responses to an open-ended, free-response, one-item measure of perceived social class: upper-class (*n* = 33), middle-class (*n* = 174), lower-class (*n* = 29), and working-class (*n* = 62); the 62 participants who identified themselves as working-class were excluded from further analysis because it was not possible to categorize them into one of the three aforementioned categories.

### 2.2. Instruments

Participants were administered a survey including demographic questions (age, ethnic identity, gender, education, self-identified social class) and items on religious/spirituality, a life satisfaction measure (SWL), and a measure of beliefs in a just world (BJW). The Satisfaction with Life Scale (SWL) is a five-item Likert-type scale developed by Diener et al. [27]. The SWL has been shown to be a valid measure of life satisfaction and includes statements such as “In most ways, my life is close to my ideal.” and “The conditions of my life are excellent”. The seven-point scale uses the following anchor points: 1 = strongly disagree, 7 = strongly agree. The Belief in a Just World Scale (BJW) [2] consists of 16 items rated on seven-point Likert scales: 1 = strongly disagree to 7 = strongly agree. Four subscales are included in the instrument: (a) distributive justice for others (DJ-others) with items such as “I feel that people usually receive the outcome they are due”; (b) procedural justice for others (PJ-others) with items such as “People are generally subjected to processes that are fair”; (c) distributive justice for self (DJ-self) with items such as “I usually receive the outcomes I deserve”; and (d) procedural justice for self (PJ-self) with items such as “People usually use fair procedures in dealing with me”. The scale has been shown to be valid and reliable in previous research, with Cronbach α for all of the subscales ≥0.89 [7,23].

Religious affiliation was measured on a binary scale that grouped religious participants (people who identified with any religion or spiritual practice) in contrast to non-religious participants (people who had no religious affiliation).

## 3. Results

### 3.1. Reliability

Mean scores, standard deviations, and Cronbach alphas are presented in Table 1. The Cronbach alphas for the scales used in the study were at 0.85 and above, suggesting acceptable reliability values. Given that 62% of the respondents completed the questionnaires on paper and 38% online, multivariate analysis on SWL and the four BJW subscales indicated *F* (5292) = 0.86, *p* = 0.511, suggesting there was no effect of participation format.

### 3.2. Demographic Characteristics and Just World Beliefs

The relationships of demographic characteristics with BJW subscales were examined using multivariate and univariate analyses. Scheffè’s post hoc test was used as required. A type 1 error probability of 0.05 was used to determine significance in all analyses. The only exception to this analytic strategy was with age, which was correlated with the BJW subscale scores.

Sex, ethnicity, age, religion/spirituality, and BJW. A multivariate analysis on the four BJW subscales revealed a multivariate *F* (4293) = 1.49, *p* = 0.204, suggesting no significant differences due to sex. Ethnicity was classified as White (*n* = 200) and non-White (*n* = 98). Initial multivariate analysis indicated a significant difference due to ethnicity on the four BJW subscales, *F* (4293) = 3.54, *p* = 0.008. The follow up ANOVA on each of the subscales indicated a significant difference between the two groups, PJ-self: *F* (1296) = 13.18, *p* = 0.000, η^2^ = 0.043 (small effect size), with Whites (*M* = 19.61) scoring higher than non-Whites (*M* = 17.54). *F* values did not reach significance at the 0.05 level for the other subscales; η^2^ values were 0.01 or lower, suggesting trivial effect sizes. Age had a small (*r* = 0.161, *p* = 0.005), but significant, correlation only with the BJW’s procedural-other subscale.

In order to explore the relationship between religion/spirituality and BJW, we classified participants into two categories: (a) those who reported an affiliation with a religion (*n* = 192) and (b) those who stated that they were neither religious nor spiritual (*n* = 106). A multivariate analysis on the four subscales revealed a significant difference due to religious affiliation/spirituality: *F* (4293) = 3.64, *p* = 0.006. Further univariate ANOVAs revealed significant differences on two subscales: (a) distributive-others: *F* (1296) = 12.82, *p* = 0.000, η^2^ = 0.042 (small effect size); people who identified with a religion/spirituality (*M* = 15.94) scored higher than people who did not identify with a religion/spirituality (*M* = 13.61); (b) procedural-others: *F* (1296) = 7.65, *p* = 0.006, η^2^ = 0.025 (small effect size); people who identified with a religion/spirituality (*M* = 15.19) scored higher than people who did not identify with a religion/spirituality (*M* = 13.61).

### 3.3. Social Class and Just World Beliefs

The participants grouped into three categories of low, middle, and upper classes were examined for differences on BJW scales. An overall significant difference was found across the four BJW subscales: multivariate *F* (12,770) = 2.04, *p* = 0.019. Further univariate ANOVAs revealed significant *F* values on the procedural subscale of both self and others: PJ-other, *F* (3294) = 3.28, *p* = 0.021, η^2^ = 0.032; PJ-self, *F* (3294) = 3.10, *p* = 0.027, η^2^ = 0.031 (small effect size). Post-hoc analysis on these variables using Scheffè’s procedure indicated a significant difference in PJ-others, with the lower-class category differing from the middle-class (upper-class *M* = 14.12; middle-class *M* = 15.22; lower-class *M* = 12.45). For the PJ-self, there were no significant pairwise differences found among the three classes (upper-class *M* = 20.61; middle-class *M* = 19.14; lower-class *M* = 17.48) at the 0.05 level, but there was a marginal difference between the upper class and lower class at the *p* = 0.077 level. Analysis for age found a positive significant relationship with the PJ-other scale (*r* = 0.161, *p* = 0.005) and the SWL (*r* = 0.125, *p* = 0.031) (see Table 2).

### 3.4. Sex/Ethnicity and Satisfaction with Life Scale

An independent sample *t*-test revealed a marginal significant difference between males (*n* = 130, *M* = 23.21) and females (*n* = 171, *M* = 24.61) on the SWL scale: *t* (299) = −1.86, *p* = 0.064, η^2^ = 0.011 (small effect size). Women reported somewhat greater satisfaction with their lives than men on average. An independent sample *t*-test revealed a significant difference between Whites (*n* = 202, *M* = 24.77) and non-Whites (*n* = 99, *M* = 22.43) on the SWL scale: *t* (299) = 2.97, *p* = 0.003, η^2^ = 0.029 (small effect size). White participants reported being more satisfied with their lives than non-white participants.

### 3.5. Religion, Social class, and Satisfaction with Life Scale

Significant differences were found between religiously affiliated (*n* = 195, *M* = 24.35) and non-affiliated (*n* = 106, *M* = 23.36) individuals on the SWL scale: *t* (299) = 1.27, *p* = 0.025, η^2^ = 0.005 (trivial effect size). The religiously affiliated reported being be more satisfied with their lives more than non-adherents. A one-way ANOVA was used to test differences among the upper, middle, and lower classes. The ANOVA indicated a significant *F* (3297) = 14.58, *p = 0*.000, η^2^ = 0.128 (medium effect size). Post-hoc analysis revealed the upper (*M* = 26.79) and middle (*M* = 25.23) classes significantly differed from the lower class (*M* = 19.4) at the 0.05 level of significance.

### 3.6. Correlation between Satisfaction with Life and Belief in a Just World Subscales

Given the large number of demographic variables tested and with most differences being not significant with small effect sizes, we did not examine the moderating effects of the demographic variables on the relationship between BJW and SWL subscales. The correlations of BJW with SWL subscales shown in Table 2 were as follows: DJ-others = 0.211, PJ-others = 0.256, DJ-self = 0.399, and PJ-self = 0.499 (all *p* = 0.000). A multiple regression analysis revealed that although the two self subscales (distributive and procedural) were significantly predictive of life satisfaction, the two other (distributive and procedural) subscales were not (see Table 3). These results are consistent with the finding by Lucas et al. [2].

## 4. Discussion

The findings support prior research suggesting that the people who believe that the world is a just place for themselves and others are also more likely to be more satisfied with their lives. The relationships were more pronounced for just world beliefs for the self than for just world beliefs for others. Furthermore, the two self-subscales (distributive and procedural) were significantly more predictive of life satisfaction than the two other subscales (distributive and procedural) in a multiple regression analysis. These findings replicate those of Lucas et al. [2] and are consistent with their theorizing that perceptions of the world being fair to oneself should be related to perceptions of life satisfaction. Lucas et al. [2] reviewed other literature that suggested that the “Belief that the world is fair to one’s self have been shown to strongly predict measures psychological adjustment and well-being” (p. 14).

Analysis of age and social class found a small positive relationship between age and procedural belief in a just world for others (PJ-others). This domain of just-world beliefs has been found to be related to social attitudes involving the evaluations of the fairness, altruistic behavior, and forgiveness by other investigators [2,4,6]. It would seem possible that with age there is an increase in a prosocial orientation. Social class analysis indicated that middle-class participants scored significantly higher on procedural belief in a just world for others (PJ-others) than lower-class participants. Oldmeadow and Fiske [28] suggested that when perceiving members of a different social class, high-status out-groups (in this case, middle-to-upper-class participants) tend to be stereotyped as competent, while low-status groups (lower-class participants) tend to be stereotyped as incompetent, which is consistent with the notion that people who have a higher belief in a just world for others may also have harsher social attitudes. The findings, though consistent with the literature, cannot be representative of the general outlook of members of these social classes. Ethnicity is an important consideration when discussing beliefs in a just world, particularly given the contemporary social and political climate—White participants scored significantly higher than non-Whites in procedural belief in a just world for the self, suggesting that the White participants were more likely to believe that they are being treated fairly. Hunt [14] suggested that research on views of the world as a just place reflects a White experience. Given the historical, political, and economic hardships faced by non-Whites in the United States, the differences in belief in a just world from this study between Whites and non-Whites are reflective of those influences that could have played a part in the difference and should be acknowledged. However, given Hunt’s [14] finding that Latinos expressed greater belief in a just world than Whites, caution is needed in generalizing to all non-white ethnic groups because the non-white group in the current study included a variety of ethnic groups.

Belief in a just world is a fundamental force in determining perceptions of and responses to unjust life events, and people believe the sources of those beliefs are God, nature, other people, self, and chance [11]. Jost et al. [12] hypothesized that religion provides an ideological basis for justifying existing social order and its institutions and arrangements that are seen as legitimate and impartial and, therefore, worthy of conforming to and preserving. The results of the study appear to be consistent with that framework inasmuch as people with a religious affiliation scored higher on both “other” (distributive and procedural) subscales than those who did not. The results also suggest that religious people may have a greater social identity and prosocial way of thinking.

The overall findings of this study, consistent with prior research, suggests that a positive relationship exists between just world beliefs and life satisfaction, although the relationship is more pronounced for BJW self subscales than other subscales. Demographic factors such as White/non-White racial identity, age, religion, and gender appear to also be related to life satisfaction, but this relationship warrants further exploration. Social class and age were also found to be related to life satisfaction and views of the world as a just place, with upper- and middle-class participants scoring higher. Religiosity was also found to be related to just-world beliefs for others, both distributive and procedural. An exploration of views of the world as a just place and well-being is an important area that warrants further investigation. In this study, the four subscales of belief in a just world indicated a significant relationship with life satisfaction. Given the political and social climate, research related to how people perceive the world is important. An awareness of such beliefs can help bridge the gaps in people’s understandings of how the world works, how people tend to think it works, and the ways in which such views may impact well-being. Such research has the potential to challenge expectations of fairness and justice in the world in the hopes of creating a more compassionate society. Continued explorations of the social mechanisms that perpetuate beliefs in the world as a just place can help foster an increased understanding of social justice and injustice.

## 5. Conclusions

The present study provides evidence that justice beliefs are predictive of life satisfaction, and thus justice beliefs may be important to one’s feelings of well-being. However, when life satisfaction was regressed on the justice belief subscales, the two beliefs subscales about justice for “self” (Procedural and Distributive) were more strongly predictive of life satisfaction than the two beliefs susbscale about justice for “others.” These findings suggest the primacy of how one perceives the world is just for self than over others in predicting life satisfaction, a finding possibly reflective of our strong individualistic values. The demographic factors of ethnicity, sex, age, and SES yielded either no significant effects or very low effect sizes when significant on certain variables with either justice beliefs or life satisfaction. Although the voluntary nature of the present study’s sampling limits the generalization of the results, the study does add to the extant literature inasmuch as it supports the results of earlier studies concerning the relationship between justice beliefs and life satisfaction.

## Figures and Tables

**Table 1:** Descriptive statistics and internal consistency reliability of the scales used in the study.

Scale	*n*	*M*	*SD*	Cronbach α
Distributive others	301	15.12	5.47	0.91
Procedural others	301	14.61	4.74	0.85
Distributive self	298	18.83	4.86	0.90
Procedural self	298	18.93	4.72	0.90
Satisfaction with life	301	24.00	6.50	0.85

**Table 2:** Correlations of age with belief in a just world (BJW) and life satisfaction measure (SWL) and between BJW and SWL.

Scale	*n*	*Age r*	*p*	*SWL r*	*p*
Distributive others	301	−0.002	0.972	0.211	0.000
Procedural others	301	0.161	0.005	0.256	0.000
Distributive self	298	−0.018	0.759	0.399	0.000
Procedural self	298	0.023	0.688	0.499	0.000
Satisfaction with life	301	0.125	0.031		

**Table 3:** Prediction of life satisfaction by BJW subscales.

Scale	*B*	*β*	*p*
Distributive others	−0.003	−0.002	0.973
Procedural others	−0.002	−0.001	0.987
Distributive self	0.294	0.223	0.003
Procedural self	0.384	0.283	0.000
